# Nutrient Intake and Physical Activity of School-Aged Children with Trisomy 21 Living in Manitoba, Canada

**DOI:** 10.3390/nu18091330

**Published:** 2026-04-23

**Authors:** Maria S. Baranowski, Carla G. Taylor, Nancy Hansen, Shahin Shooshtari

**Affiliations:** 1 College of Community and Global Health, University of Manitoba, Winnipeg, MB R3T 2N2, Canada; shahin.shooshtari@umanitoba.ca; 2 St.Amant Research Centre, Winnipeg, MB R2M 3Z9, Canada; 3 Department of Food and Human Nutritional Sciences, University of Manitoba, Winnipeg, MB R3T 2N2, Canada; carla.taylor@umanitoba.ca; 4 Albrechtsen Research Centre, St. Boniface Hospital, Canadian Centre for Agri-Food Research in Health and Medicine, Winnipeg, MB R2H 2A6, Canada; 5 Educational Administration, Foundations & Psychology, University of Manitoba, Winnipeg, MB R3T 2N2, Canada; nancy.hansen@umanitoba.ca

**Keywords:** dietary assessment, dietary intake, food and beverage consumption, nutrient intake, physical activity, children, Trisomy 21, Down syndrome

## Abstract

**Background:** Children and adults with Trisomy 21 are more likely to develop nutrition-related conditions and diseases. The nutrition-related health of Canadians with Trisomy 21 is unknown. We aimed to determine the nutrient intake and physical activity of school-aged children with Trisomy 21 in Manitoba, Canada. **Methods:** Mothers of 14 school-aged children (*n* = 7 female, average age 9 years old) with Trisomy 21 completed a 24 h dietary recall and a survey that included questions about their children’s nutrition and physical activity. Nutrient intake analysis was conducted to compare food and beverage consumption with dietary guidelines and nutrient recommendations. Data were analyzed descriptively. **Results:** Most children with T21 included in this study consumed an adequate average intake of daily protein, carbohydrate, and iron; an inadequate average intake of daily dietary fibre and calcium; and an excessive average daily intake of added sugars and saturated fat. Notably, all children consumed inadequate vitamin D and excessive sodium. Most children consumed a dietary supplement (10/14), engaged in moderate-intensity physical activity (10/14), and were active for more than 60 min per day (12/14). **Conclusions:** Most children with Trisomy 21 included in this study met daily physical activity recommendations. However, despite a variety of foods reportedly consumed across all food groups, nutrient intake among school-aged children with Trisomy 21 included in this study was mixed, as both deficiencies and excessive amounts of some nutrients were observed. There is a need to improve the nutrient intake of children with Trisomy 21 to reduce their risk of developing nutrition-related conditions and diseases.

## 1. Introduction

Down syndrome (DS) is a naturally occurring chromosomal arrangement associated with chromosome 21 and present in 1 in every 750 live births in Canada, based on data collected between 2005 and 2013 [1]. As of 2020, there are approximately 22,367 people with DS in Canada [2]. Trisomy 21 (T21) is the most common form of Down syndrome [3], wherein there is an extra copy of chromosome 21.

Individuals with T21 are at increased risk of developing nutrition-related conditions and diseases such as overweight or obesity [4,5], diabetes [6], sarcopenia [7], and Alzheimer’s disease [8,9,10]. Despite the identification of several dietary patterns that are associated with a reduced risk of nutrition-related conditions and diseases, including cognitive impairment [11] and Alzheimer’s disease [12], in people without T21, this same association has not been studied in people with T21. This is unsurprising given that people with intellectual and developmental disabilities (IDD), a term that is commonly used to categorize T21, have not been historically included and accurately represented in health and nutrition research [13]. Researchers in the UK recently reported that less than 1% of research funding was allocated for genetic syndromes, including T21. Advocacy for the consideration of people with complex needs, such as those with IDD, in research and policy has been stated [14], including the reasoning that optimal nutrition is essential to those who live with disability [15]. Indeed, the exclusion of people with T21 in health and nutrition reflects ableism within health and research practice and society at large.

There are currently no specific nutrition recommendations or guidance for Canadians with T21, although DS is included among a list of ‘other diagnoses’ in the Canadian Paediatric Society (CPS) practice point for the nutritional evaluation of the neurologically impaired child [16]. There is no clear directive in the CPS practice point to provide a referral to professional nutrition services; rather, clinicians are advised to exercise clinical judgement when determining nutrient needs, considering that only limited, and possibly inaccurate, nutrition data are available. Notably, it is questionable whether these clinicians are adequately trained to conduct comprehensive nutritional assessments or provide person- or family-centred nutrition counseling [17]. Indeed, improving the nutrition education within medical curricula, and building partnerships between physicians and dietitians to facilitate the referral process, has been recommended by others [18]. However, even among Canadian dietitians, there appears to be a lack of training and resources to support their provision of nutrition knowledge and care to persons with disabilities [19].

In Canada, no study has previously examined the nutrition-related health of children with T21, and there is currently no monitoring of the nutritional status of this population. Given the lack of basic knowledge about nutrition-related health among Canadians with T21, as a first step, we aimed to determine the nutrient intake and physical activity of school-aged children between the ages of 6 and 12 years old with T21 living in Manitoba. This research is significant not only for its novelty, but also because the findings can inform meaningful and relevant *preventative* nutrition and healthcare practice for Canadians with T21. In turn, this can improve their nutrition-related health, reduce their risk of developing nutrition-related conditions and diseases, and facilitate their attainment of health and nutrition equity.

## 2. Materials & Methods

As part of a cross-sectional exploratory descriptive mixed-methods research study, food and beverage consumption and physical activity data were collected using a 24 h dietary recall and survey instrument. The study was conducted in the province of Manitoba, Canada. Approximately 1.3 million people live in Manitoba, and just over 250,000 are children between the ages of 0 and 14 years old [20]. Approximately 36% of Manitobans between the ages of 35 and 64 years old reside in a rural setting, and approximately 38% reside in an urban setting [20]. Approximately 18.5% of Manitobans self-identify as Indigenous, 90% are Canadian citizens, and 20% are immigrants.

The study population consisted of school-aged children with T21 between the ages of 6 and 12 years old, with or without any siblings, living in Manitoba. The food choices of younger children are more likely to be influenced by the food purchasing and food preparation choices of their parents and caregivers. The food choices of older children may be more influenced by their peers or other food environments. Given the potential differences in the factors that influence food choices among younger and older children, only younger children were included in this study. Parents or caregivers consented to be study participants in this research study and to share information on behalf of their child. A total of 14 children and 14 parents or caregivers were included in this study. Given the novelty of the study, as there is no current data surveillance about the nutrition-related health of Canadians with T21, the sample size was considered adequate and feasible within the timeframe of a doctoral graduate program. Ethical approval was granted from the University of Manitoba Health Research Board on 19 August 2024, #HS26565 (H2024:227).

Recruitment was planned to attain a convenience sample of study participants who were parents and caregivers of children with T21 living in Manitoba. No exclusion criteria were applied in an effort to recruit a diverse study sample. Recruitment strategies were informed by parent engagement research partners, who were consulted before the research study began. Multiple advocacy organizations were contacted to assist with distributing information about the study. Those who agreed were provided with a one-page electronic research summary for their use to share with their networks. The first author’s positionality as the researcher and interviewer, a woman, a parent of three children (including one with T21), a rural Manitoba resident, a parent advocate for inclusion, a dietitian, and a graduate student, was shared with all study participants before beginning data collection. Reflexive memos were written by the same after every data collection meeting with study participants to identify and mitigate any personal biases.

Information about the study was reviewed with study participants before consent was requested and provided, and before any data were collected. Verbal consent to conduct and record the virtual data collection meeting was requested before beginning data collection. Food and beverage consumption and data about physical activity were collected using a 24 h dietary recall and a survey instrument. The survey instrument included questions about dietary supplement usage and physical activity level and intensity. Specifically, the questions were: (1) Does your child currently take a multivitamin or supplement of any type? (2) Approximately how many minutes of physical activity does your child engage in on a daily basis (less than 60 min, more than 60 min, I don’t know)? and (3) Does your child engage in low, moderate, or high intensity when they are physically active? Data collection occurred virtually using Microsoft Teams or Zoom between December 2024 and February 2025. The type and content of the data collection tools were informed by parent engagement researchers, who were consulted before beginning the research study.

The process for conducting the 24 h dietary recall in the Canadian Community Health Survey [21] was followed in this research study. The selection of the 24 h dietary recall as a dietary data collection tool was informed by parent engagement researchers. A 24 h dietary recall was considered sufficient and representative to determine *baseline* data about the types and amounts of foods and beverages among a small sample of children with T21. Further, the 24 h dietary recall did not require parents and caregivers to complete any work before the data collection meeting. Given the age and potential needs of their children, the goal was to select a data collection tool that minimized the burden of participating in the research study. The scheduling of data collection meeting times with study participants ensured that the 24 h dietary recall was reflective of the child’s intake on a previous Canadian school weekday, and not during regularly scheduled spring or winter breaks, during a statutory or national holiday, or during the summer months. Probing questions were used to ensure accuracy and completeness of data. The survey instrument was designed to collect information about several nutrition-related factors; however, only data pertaining to dietary supplement usage and physical activity are presented in this paper. The survey instrument was developed by the first author and reviewed by the co-authors for face validity. An honorarium was provided to each study participant at the conclusion of the data collection meeting. Transcripts of each data collection meeting were recorded using Zoom or Microsoft Teams and saved in a secure, single-user, multi-factor-authenticated online university account.

Analysis of nutrient intake based on food and beverage consumption data collected using the 24 h dietary recall was conducted using FoodFocus Version 4.2 (FoodFocus, Winnipeg, MB, Canada). FoodFocus is a nutrition analysis software that contains nutrient data for nearly 6000 foods, incorporated from the 2015 Canadian Nutrient File (published by Health Canada), and compares food and beverage consumption to age-specific national nutrient recommendations such as the dietary reference intakes (DRIs) and Acceptable Macronutrient Distribution Range (AMDR) [22,23]. Afterwards, the average intake for each nutrient for the entire group of children was calculated, as was the proportion of the study population that consumed intakes that were more than, within, or less than the DRIs. Food lists from the 24 h dietary recall data collection tool were also reviewed and categorized according to food group or type by the first author, a registered dietitian. Specifically, foods were divided into the following groups/types: whole grains, other grain products, meat products, meat alternatives, dairy products, fruits and vegetables, snack foods, other foods, and restaurant meals. The most common reported beverages consumed were also summarized. Then the average number of servings of different food groups/types was calculated at the aggregate level. Descriptive statistics were used to analyze dietary supplement usage and physical activity data collected using the survey instrument using Microsoft Office Excel Version 16.77.1 (Microsoft, Redmond, WA, USA).

## 3. Results

Parents of 14 school-aged children between the ages of 7 and 12 years old with T21 were included in the study (*n* = 14). The average age of children with T21 was 9 years, and half were female (*n* = 7). All parents who consented to participate in this research study were moms. Most of the moms (93%; *n* = 13) were between 31 and 50 years old. None of the moms had moved to Canada within the previous year, and just over half (53%; *n* = 8) resided in an urban setting within Manitoba. The majority of the moms (86%; *n* = 12) had lived in Manitoba for over 31 years at the time of data collection. Slightly over two thirds of the moms (79%; *n* = 11) described themselves as Caucasian, and only three (21%; *n* = 3) self-identified as Indigenous. Almost two thirds (77%; *n* = 10) of the moms reported an annual household income greater than CAD 100,000. Characteristics of the moms are summarized in Table 1.

### 3.1. Food and Beverage Consumption

Food and beverage consumption data obtained via the 24 h dietary recall completed by each participating mom on behalf of their child with T21 were descriptively analyzed and categorized by food type. Meat alternatives consumed by children with T21 in this study included mostly peanut butter and eggs and, rarely, beans, lentils, or seeds. Also, dairy products consumed tended to be mostly cheese and flavoured yogurt choices, followed by milk. The remaining food items were organized into additional categories such as whole grains, snack foods, restaurant meals, and other foods. Whole grains consumed by children with T21 in this study were oatmeal and popcorn. Snack foods included crackers, applesauce, granola bars, and pretzels. Other foods included condiments, chocolate, chips, juice, and dessert.

### 3.2. Canada’s Food Guide

When applicable, serving sizes from the 2011 version of Eating Well With Canada’s Food Guide [24] were used to estimate the number of servings from specific food groups, such as grain products, meat products and alternatives, dairy products, and fruits and vegetables. When serving sizes were not applicable, for example, for snack foods or restaurant meals, frequency of consumption was tracked. A summary of the types of food consumed daily by children with T21 in this study is presented in Table 2.

Among the children with T21, the average number of servings of whole grains (0.79 ± 1.25) was low. The average number of servings of grain products was the highest (3.54 ± 1.76), followed by fruits and vegetables (3.21 ± 2.80), dairy products (2.46 ± 1.70), and meat products (1.29 ± 0.83). Notably, the average number of servings of meat alternatives (peanut butter, eggs, lentils, beans, seeds) was nearly as high as the average number of servings of meat products (1.21 ± 1.42), which aligns with the current version of Canada’s Food Guide [25], which recommends greater consumption of plant-based sources of protein foods. Among the children with T21, the frequency of consumption of ‘other’ foods (condiments, chocolate, chips, juice, dessert) was relatively high (4.18 ± 2.38), that of snack foods (crackers, applesauce, granola bars, pretzels) was relatively moderate (1.71 ± 1.54), and that of restaurant meals was low (0.21 ± 0.43).

**Figure 1:**
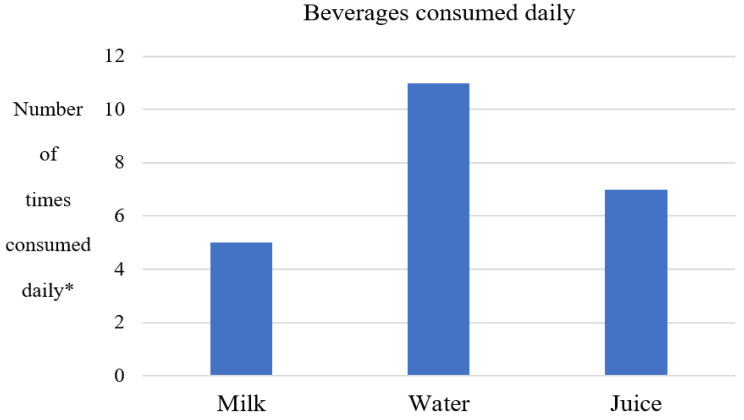
Summary of the frequency of beverage consumption by type among children with T21. * Total number of times beverages were reported by study population. Quantity of each reported beverage choice was variable, sometimes immeasurable (‘sips’), and sometimes unknown (when beverages were consumed outside of home environment).

Beverages consumed by children with T21 in the study, based on data collected from the 24 h dietary recall, included water, juice, and milk (Figure 1). Nearly all children with T21 (*n* = 11) reported drinking water daily. Half of the children with T21 (*n* = 7) consumed juice daily, and less than half (*n* = 5) consumed milk daily. Notably, no soft drink consumption was reported for children with T21 in the study population.

### 3.3. Dietary Reference Intakes

Dietary reference intakes (DRIs) were used to assess food intake variety based on the adequacy of selected nutrient intakes. Among the children with T21 in this study, 86% (*n* = 12) had a daily protein intake within the AMDR, 71% (*n* = 10) consumed a daily carbohydrate intake within the AMDR, and 50% (*n* = 7) consumed a daily fat intake within the AMDR (Table 3). Notably, daily fat intake was below the AMDR for 29% (*n* = 4) of children with T21 included in this study. Additionally, as an indicator of whole grain and fruit and vegetable consumption, 21% (*n* = 3) of children with DS consumed an Adequate Intake (AI) of total dietary fibre (Table 3). Notably, daily dietary fibre intake was below the AI for 79% (*n* = 11) of children with DS included in this study (Table 3).

Nutrient intakes, obtained from the analysis of food and beverage consumption from the 24 h dietary recall, were compared to applicable age- and sex-specific recommended dietary reference intakes using FoodFocus. A summary of selected daily average nutrient intakes and their comparison to dietary reference intakes is presented in Table 4. Among the micronutrients, iron was consumed in adequate amounts by 93% (*n* = 13) of the children with DS in this study. However, no children with DS consumed the recommended amount of vitamin D, less than half (43%; *n* = 6) consumed the recommended amount of total energy, and 71% (*n* = 10) did not consume the recommended amount of calcium or fibre. Conversely, over half (57%; *n* = 8) of the children with T21 consumed more than the recommended amount of total energy, 71% (*n* = 10) consumed more saturated fat and added sugars than recommended amounts, and all (100%; *n* = 14) children with T21 consumed more sodium than the dietary guidelines suggest.

### 3.4. Dietary Supplement Usage

Daily dietary supplements were provided by parents to 71% (*n* = 10) of children with T21 included in this study. The most commonly consumed daily dietary supplement was a multivitamin, which was reportedly provided to 80% (*n* = 10) of children with T21 who consumed a daily dietary supplement. Other children who consumed a daily dietary supplement (2/10) consumed something other than a multivitamin (not reported here to protect the identity of the children).

### 3.5. Physical Activity

Approximately 86% (*n* = 12) of children with T21 engaged in more than 60 min of physical activity per day, and for 71% (*n* = 10), their physical activity was reportedly of moderate intensity (versus low or vigorous). In this study, moderate intensity was characterized as can talk but not sing, whereas vigorous intensity was characterized as cannot say more than a few words before pausing for breath. Notably, no moms reported that their child with T21 engaged in vigorous-intensity physical activity.

## 4. Discussion

Children with T21 included in this study consumed (1) an adequate average number of daily food servings of meat products and meat alternatives and grain products, which aligned with an adequate average intake of daily protein, carbohydrate, and iron; (2) a low average number of daily food servings of fruits and vegetables, which aligned with an inadequate average intake of daily dietary fibre; (3) fewer daily servings of vitamin D-fortified milk compared to daily servings of yogurt and cheese, and low consumption of other dietary sources of vitamin D, which aligned with an inadequate average intake of daily vitamin D and calcium; and (4) frequent consumption of snacks or ‘other’ foods, which aligned with excessive average daily intakes of added sugars, saturated fat, and sodium. Notably, the average number of daily servings of foods from food groups was comparable to that observed in Canadian children of similar ages without T21 [26]. Furthermore, children with T21 included in this study consumed a variety of different types of foods, even if not always in adequate amounts, which corresponds with the varied food types consumed by children with DS reported by other researchers [27].

Our findings are similar to the pattern of food types reported among children with T21 by Gruszka & Wlodarek [28] regarding low fibre, fruit and vegetable, and whole grain intake, but different in the observed amounts of dairy and meat products consumed. Dietary fibre intake among the children with T21 included in this study did not meet DRI recommendations for 79% (*n* = 11) of participants. Further, inadequate fibre intake among children with DS has also been reported in Brazil [29], Turkey [30], Saudi Arabia [31], and the United States [32]. The daily average dietary fibre intake among the children with T21 included in this study was 19 g, which is the same as what has been observed among children with DS living in Turkey (19 g) [30], less than what has been observed among children with DS in Brazil (23 g) [29], and greater than what has been observed among children with DS in Greece (9 g) [33], Saudi Arabia (12 g) [34], and among Canadian children without DS (17 g and 15 g for boys and girls, respectively, between the ages of 9 and 13 years) [35]. Promotion of, and access to, fibre-containing foods to increase their consumption among children with T21 may be necessary to improve fibre intakes. Caregivers responsible for feeding children with T21 in home, school, and extracurricular settings may have a role in ensuring there is access and exposure to these types of foods so that children are both familiar with and inclined to consume them. Healthcare providers may encourage the provision of fruits and vegetables and whole grains to children with T21 and provide recommendations to caregivers about how to prepare and meaningfully incorporate these foods. Further, policymakers may ensure that these foods are accessible and affordable for all families.

The children with T21 included in this study consumed an average daily intake of 283 g of carbohydrate, which was higher than intakes reported among children with DS living in Brazil (280 g) [29], Saudi Arabia (247 g) [34], and Turkey (208 g) [30], but comparable to findings about carbohydrate intake (as a percentage of total energy) of American children with DS [32]. Compared to larger sample sizes (*n* = 1842) of Canadian children without T21 between the ages of 9 and 13 years old, the children with T21 included in this study consumed comparable amounts of protein, fat, and carbohydrate and fell within the AMDR [35]. A closer look at the carbohydrate-containing foods consumed most often among children with T21 may help identify those that are less nutritious and higher in added sugar or sodium, and lower in fibre. The provision of nutrition knowledge and resources (for example, fact sheets, recipes), a commitment to increasing food literacy and food skills across ages, and other tools to encourage the selection of more nutritious foods (for example, front-of-package labelling) may guide the consumption of more nutrient-dense carbohydrate-containing foods.

The children with T21 included in this study consumed a daily average energy intake of approximately 2100 kcal, which was higher than energy intakes reported among children with DS living in Turkey (approximately 1800 kcal/day) [30], Brazil (approximately 1800 kcal/day) [29], and Saudi Arabia (approximately 1700 kcal/day) [34]. However, while the daily average energy intake of children with T21 in this study was reportedly higher than Canadian female children without DS, their intakes were lower than Canadian male children without DS between the ages of 9 and 13 years old [35]. Children with T21 and their caregivers may benefit from access to healthcare providers who are able to provide meaningful nutrition knowledge and care about how to manage energy intakes in relation to individual growth and development, physical activity levels, and energy requirements. This may be particularly important given that among the children with T21 included in this study, snack foods (crackers, applesauce, granola bars, pretzels) were consumed on average 1.7 times per day, and ‘other’ foods (condiments, chocolate, chips, juice, dessert) were consumed on average 4.2 times per day. Snack and ‘other’ foods usually contain higher amounts of nutrients that provide greater amounts of energy (calories) and are typically recommended *to limit*, such as added sugar, which is reflected in the observed excessive intake of added sugar among 79% of children with DS included in this study compared to amounts determined as ‘ideal’ by the American Heart Association [36]. However, the daily average intake of added sugars among the children with T21 included in this study (approximately 41 g) was *less than* the daily limit recommended by Diabetes Canada (50 g) [37]. Notably, added sugar intake of children with T21 has not been reported elsewhere.

The average daily intake of sodium among the children with T21 included in this study was greater than recommended amounts (1000–1200 mg/day), at 2852 mg, which was lower than intakes reported among children with T21 living in Turkey (3164 mg) [30] and in Brazil (3209 mg) [29], but higher than intakes reported among Canadian children without T21 (2740 mg and 2410 mg for boys and girls aged 9–13 years old, respectively) [38]. Notably, higher-than-recommended sodium intakes have been observed among *apparently healthy* children globally, which is concerning since excessive intake is associated with high blood pressure, cardiovascular diseases, osteoporosis, diabetes, and cancer [39]. There are several different types of foods that may contribute to higher-than-recommended sodium amounts. Sharing information and strategies with parents and caregivers of children with T21 about how to identify lower-sodium foods at the point of purchase (for example, by reading nutrition labels and comparing products), or how to prepare lower-sodium meals at home (for example, by replacing higher-sodium ingredients with alternatives), may reduce the consumption of sodium among these children.

Among the children with T21 included in this study, daily total fat intake as a percentage of total energy was within recommended ranges for only half (*n* = 7) of the children, despite a high number of times ‘other’ and snack foods were consumed, which tend to contain higher amounts of fat. It may be of concern that among the children with T21 included in this study, approximately one third (*n* = 4) did not meet the recommended intake for fat, an important macronutrient required for optimal growth and development during childhood. Compared to Canadian children between the ages of 9 and 13 years old without T21 [35], who consumed approximately 33.4% of total energy from fat, the children with T21 included in this study consumed less total fat as a percentage of total energy intake per day (32.1%), but both were still within the AMDR. Children with T21 included in this study consumed on average approximately 81 g of fat per day, which is more than the average amount consumed by children with DS living in Turkey (73 g) [30], Brazil (53 g) [29] and Saudi Arabia (53 g) [34]. Notably, 21% (*n* = 3) of the children with T21 in this study consumed more than the recommended amount of total fat based on their food and beverage consumption. High intakes of fat over time are associated with increased risk of cardiovascular disease [40], particularly higher intakes of saturated fat, which were observed among 71% (*n* = 10) of the children with T21 in this study. Nutrition knowledge shared with parents and caregivers of children with T21 about the different types of fat and their outcomes, such as the need for essential fatty acids as well as the need to balance the intake of some types (and the total amount) of fat to mitigate disease risk, may be helpful for informing decisions regarding purchasing and preparing fat-containing foods.

Adequate protein intake observed among the children with T21 included in this study align with findings reported by other researchers in Greece [33] and the United States [32]. Children with T21 included in this study consumed on average approximately 73 g of protein a day, which was higher than average amounts reported among children with DS living in Brazil (68 g) [29], Turkey (71 g) [30], and Saudi Arabia (55 g) [34]. Notably, children with T21 included in this study consumed on average the same number of servings of meat alternatives as meat products (1 serving per day of each), which aligns with current dietary guidance to consume more plant-based protein [25]; adequate amounts of meat alternatives were also observed among children with DS living in Italy [41].

Furthermore, iron intake levels were within recommended ranges for most of the children with T21 included in this study (93%; *n* = 13), which aligns with the observation of adequate intake of meat products. This is important, given that supplementation of this mineral may be recommended in some children with DS who experience sleep problems and have low ferritin concentrations, which is an indicator of iron storage in the blood [42]. Likewise, Grammatikopoulou and colleagues [33] reported that 75% of their study sample, consisting of children with DS between the ages of 6 and 9 years old living in Greece, met the daily dietary reference intake for iron. The daily average intake of iron among the study population was 15 mg, which was higher than intakes reported for children with DS living in Turkey (10 mg) [30], Brazil (14 mg) [29], and the United States (10 mg) [32], but lower than that for children with DS living in Saudi Arabia (25 mg) [34]. Children with T21 included in this study consumed a higher daily average of iron than Canadian children without T21 (approximately 14 g and 12 g for boys and girls, respectively, between the ages of 9 and 13 years) [35].

Children with T21 included in this study consumed an average of 2.5 servings of dairy products per day, which is within the recommended number of servings suggested by older versions of Canada’s food guide for children between the ages of 5 and 13 years old [24]. However, despite the apparent adequate daily intake of dairy products, daily nutrient levels of calcium were less than DRIs for 71% (*n* = 10), likely due to consumption of cheese and yogurt dairy products being reported more often than that of milk, which contains greater amounts of calcium per serving size. The daily average intake of calcium among the children with T21 included in this study was 899 mg, which is higher than amounts reported among children with DS in Brazil (688 mg) [29], Turkey (840 mg) [30], Saudi Arabia (515 mg) [34], and the United States (897 mg) [32]. The children with T21 included in this study consumed a daily average intake of calcium comparable to that of Canadian children without T21 (approximately 1100 milligrams and 900 milligrams for boys and girls, respectively, between the ages of 9 and 13 years) [35]. Dairy allergy and lactose intolerance may need to be considered as potential factors that may influence food intake among children with T21 when promoting the consumption of calcium-rich food choices.

Levels of vitamin D intake from food and beverage consumption were below recommended intakes for all 14 children with T21 included in this study. This is not surprising given that vitamin D-fortified milk and fatty fish are among the few food sources of dietary vitamin D [43], and neither of these were consumed in notable amounts by the children with DS included in this study. The average daily intake of vitamin D among children with DS included in this study was 115 IU, which is lower than the average daily intake levels of vitamin D observed among children with T21 living in Turkey (approximately 222 IU) [30], and those of Canadian children *without* T21 (228 IU and 188 IU for boys and girls aged 9–13 years old, respectively), but higher than intakes reported among children with T21 living in Brazil (94 IU) [29]. While vitamin D deficiency is reported among half the Canadian population between the ages of 3 and 79 years old [44], particular attention to vitamin D may be important among children with T21, as vitamin D plays an important role in bone development and maintenance, reducing inflammation, immune function, and glucose metabolism [45]. It is concerning that none of the children included in our study received adequate vitamin D from their food and beverage consumption, whereas 75% of Canadian children between the ages of 6 and 11 years old without T21 reportedly have adequate vitamin D levels, based on data collected between 2016 and 2019 [44]. Caregivers of children with T21 may benefit from the provision of knowledge about the importance of vitamin D for their child’s health, as well as how to attain adequate amounts of vitamin D from food and beverage consumption.

Among the children with T21 included in this study, water, juice, and then milk were the most frequently consumed beverage choices. Compared to other beverages choices (for example, soda), juice and milk may be considered more nutritious, as they are a source of some nutrients (for example, vitamin C, calcium, vitamin D). Notably, none of the children with T21 included in this study consumed carbonated sugar-sweetened soft drinks. Fluid intake of children with T21 has not been described in detail in existing literature, although Magenis and colleagues reported low water intake among children with T21 living in Brazil [29], and a greater variety of sugar-containing beverages was reported among Canadian children without T21 [46]. For example, Warren and colleagues [46] collected data about the consumption of sugary drinks (described as beverages with free sugars), sugar-sweetened beverages (described as beverages with added sugars), and 100% juice (beverages with natural sugars).

Among the children with T21 included in this study, daily dietary supplementation was common (71%; *n* = 10). This finding is consistent with findings of previous studies of children with T21 [47]. However, the rate of supplementation among the children with DS included in this study (71%) was much higher than the reported rate for children with T21 living in the United States (approximately 38%) [47] and in Turkey (approximately 15%) [30]. For the majority (80%) of dietary supplement users among the children with DS included in this study, the dietary supplement provided was a daily multivitamin, which may have helped to address inadequate dietary intakes of nutrients such as calcium and vitamin D.

Approximately 71% (*n* = 10) of the children with T21 included in this study reportedly met the recommended physical activity level of at least 60 min per day at the time of the study [48]. Their activity level was described as mostly moderate intensity by their moms. Similar findings were published by researchers in Japan [49], while researchers in Italy reported higher levels of daily physical activity among children with T21 (minimum 2 h per day) [41]. Conversely, among Canadian children in the general population between the ages of 5 and 17 years old, only 40% met the physical activity guidelines [50]. Future research among a greater sample size may consider analyzing nutrient intake by level and/or intensity of physical activity. While a greater proportion of the children with T21 included in this study engaged in physical activity compared to what has been observed among the child population in Canada at large, the intensity of their physical activity may be improved from low–moderate to high. Furthermore, increasing the number of opportunities for children with T21 to engage in physical activity, or prescribing structured physical activity [51], may help children with DS and their parents meet and maintain an active lifestyle.

There are several strengths of our research study. To our knowledge, this is the first Canadian research study to include children with T21 and determine their nutrient intake, dietary supplement usage, and physical activity levels. There is currently only one national survey on children and youth in Canada (the Canadian Health Survey on Children and Youth), which collects a wide range of health data; however, it did not obtain data about nutrition-related health. Additionally, although children with some type of long-term health condition/disability are specified (e.g., learning disability or disorder, autism spectrum disorder, or Fetal Alcohol Spectrum Disorder), they have not included T21, which prevents us from learning about their health across Canadian jurisdictions. To improve data availability for health assessment and surveillance, it is pertinent to add questions about nutrition-related health to such surveys and specify T21 as one of the types of long-term health conditions. Second, this study is an example of an inclusive nutrition research study that aimed to learn more about children with T21, who have not been historically included or accurately represented in research. Third, parent engagement research partners were consulted before beginning the research study, and their feedback was used to inform several aspects of the research study.

This study presents several limitations that should be considered when interpreting the findings. First, the small sample size (*n* = 14) limits the generalizability of the results and reduces statistical power. Second, dietary intake was assessed using a single 24 h dietary recall, which may not accurately reflect habitual dietary patterns and is subject to recall bias. Third, the absence of a control group prevents comparisons with children without Trisomy 21, limiting the ability to determine whether the observed nutritional patterns are specific to this population. Additionally, the cross-sectional and descriptive design precludes any causal inferences between dietary intake and health outcomes. Finally, potential confounding factors such as socioeconomic status, parental education, and clinical comorbidities were not fully explored. For example, some findings may have been pronounced among lower-income households. Future studies with larger samples and more robust methodologies are needed to validate these findings.

## 5. Conclusions

The nutrient intake of children with T21 living in Manitoba, Canada, does not meet several dietary recommendations. Based on the findings from our research study, we recommend monitoring food and beverage consumption and physical activity levels of children with T21 to inform and improve practice guidelines among health care providers who serve them. A greater understanding of the nutrition-related needs of children with T21 may improve the provision of meaningful nutrition knowledge and care to these children and their access to the same, and reduce their risk of developing nutrition-related conditions and diseases.

## 6. Future Perspectives

Future research may expand the current findings by including larger and more diverse populations of children with Trisomy 21 to improve generalizability. Longitudinal studies are needed to better understand the relationship between dietary patterns, nutrient intake, and long-term health outcomes. For example, future studies that aim to determine insulin resistance, inflammation, or gut microbiota among children with T21 can help better understand their risk of cardiovascular and metabolic disease. Further, the use of more comprehensive dietary assessment tools, such as multiple-day recalls, may provide a more accurate representation of habitual intake. Additionally, future studies may explore the role of nutritional interventions in improving health outcomes in this population, including strategies targeting micronutrient deficiencies and excessive sodium intake. Ultimately, integrating nutritional assessment into routine clinical care for individuals with Trisomy 21 may contribute to more personalized and effective health management strategies.

## Figures and Tables

**Table 1:** Characteristics of the parent study participants.

	Proportion *n* (%)
Relationship to the child	
Mother	14 (100%)
Father	0 (-)
Valued nutrition to child’s overall health	
Yes	14 (100%)
No	0 (-)
Age of parent	
Between 31 and 50 years old	13 * (93%)
Moved to Canada within the past year	
Yes	0 (-)
No	14 (100%)
Residence in Manitoba	
Urban	8 (57%)
Rural	6 (43%)
Ethnicity **	
Caucasian	11 (79%)
Indigenous	3 (21%)
Other	2 (14%)
Annual household income ***	
Less than CAD 50,000	0 (-)
Between CAD 50,000 and CAD 100,000	3 (21%)
Greater than CAD 100,000	10 (71%)

* Age of remaining study participant not presented to protect their anonymity. ** Multiple self-reported responses permitted. *** Distribution was based on answers from 13 study participants.

**Table 2:** Summary of daily food types consumed by children with T21.

Food Type	Average Number of Servings Consumed per Day * Mean ± SD (Range)
Whole grains **	0.79 ± 1.25 (0, 4)
Grain products	3.54 ± 1.76 (0, 5)
Meat products	1.29 ± 0.83 (0, 3)
Meat alternatives ***	1.21 ± 1.42 (0, 5)
Dairy products ^+^	2.46 ± 1.70 (0, 5)
Fruits and vegetables	3.21 ± 2.80 (0, 8)
Snack foods ^++^	1.71 ± 1.54 (0, 4)
Other foods ^+++^	4.18 ± 2.38 (0, 8)
Restaurant meals	0.21 ± 0.43 (0, 1)

* According to Eating Well With Canada’s Food Guide servings, if applicable (not all food types have recommended servings). ** Oatmeal and popcorn. *** Mostly peanut butter and eggs; beans and lentils (*n* = 2); seeds (*n* = 1). ^+^ Cheese, yogurt, and milk. ^++^ Crackers, applesauce, granola bars, pretzels. ^+++^ Condiments, chocolate, chips, juice, dessert.

**Table 3:** Daily macronutrient intake of children with T21 compared to dietary reference intakes (DRIs).

Macronutrient	Average % Energy (Range)	AMDR * % Energy	More than DRI *n* (%)	Meets DRI *n* (%)	Less than DRI *n* (%)
Protein	14 (6, 20)	10–30	0 (-)	12 (86%)	2 (14%)
Carbohydrate	54 (36, 68)	45–65	2 (14%)	10 (71%)	2 (14%)
Fat	32 (22, 57)	25–35	3 (21%)	7 (50%)	4 (29%)

* DRI Acceptable Macronutrient Distribution Range (AMDR).

**Table 4:** Daily nutrient intake of children with T21 compared to dietary reference intakes (DRIs).

Nutrient	Average per Day Mean ± SD (Range)	DRI	More than DRI *n* (%)	Meets DRI *n* (%)	Less than DRI *n* (%)
Total energy	2119 ± 606 kcal (1193, 2961)	1300–2400 kcal/day *	8 (57%)	0 (-)	6 (43%)
Protein	73 ± 29 g (27, 135)	30–222 g/day *	0 (-)	12 (86%)	2 (14%)
Fat	81 ± 41 g (30, 167)	33–115 g/day *	3 (21%)	7 (50%)	4 (29%)
Saturated fat ^+^	10 ± 3% kcal (4, 16)	-	10 (71%)	4 (29%)	0 (-)
Trans fat ^++^	1 ± 1 g (0, 5.7)	-	-	-	-
Carbohydrate	283 ± 67 g (174, 398)	134–481 g/day *	2 (14%)	10 (71%)	2 (14%)
Added sugars ^+^	8 ± 4% kcal (1, 13)	-	10 (71%)	4 (29%)	0 (-)
Fibre	19 ± 7 g (6, 29)	18–34 g/day **	0 (-)	3 (21%)	11 (79%)
Sodium	2852 ± 935 mg (1305, 4396)	1200–1500 mg/day **	14 (100%)	0 (-)	0 (-)
Calcium	899 ± 423 mg (302, 1541)	1000–1300 mg/day *	0 (-) ^^^	4 (29%)	10 (71%)
Iron	15 ± 7 mg (6, 33)	8–10 mg/day *	0 (-) ^^^	13 (93%)	1 (7%)
Vitamin D **	115 ± 76 IU (10, 244)	600 IU/day *	0 (-)	0 (-)	14 (100%)

*n* = 14. * Daily intake compared to DRI AMDR. ** Daily intake compared to DRI Adequate Intake (AI). ^+^ Compared to American Heart Association (AHA) ‘above ideal’ guideline. ^++^ No recommended daily intake level established. ^^^ No daily intake exceeded the Tolerable Upper Limit (2500 mg/day for calcium and 40 mg/day for iron for female and male children 4–13 years).

## Data Availability

The raw data supporting the conclusions of this article will be made available by the authors on request due to confidentiality and anonymity of the small study size.
